# Microwatt Volatile Optical Bistability via Nanomechanical Nonlinearity

**DOI:** 10.1002/advs.202300042

**Published:** 2023-04-26

**Authors:** Dimitrios Papas, Jun‐Yu Ou, Eric Plum, Nikolay I. Zheludev

**Affiliations:** ^1^ Optoelectronics Research Centre and Centre for Photonic Metamaterials University of Southampton Highfield Southampton SO17 1BJ UK; ^2^ Centre for Disruptive Photonic Technologies, SPMS, TPI Nanyang Technological University Singapore 637371 Singapore

**Keywords:** metamaterials, nanomechanics, nanophotonics, nonlinear optics, plasmonics

## Abstract

Metastable optically controlled devices (optical flip‐flops) are needed in data storage, signal processing, and displays. Although nonvolatile memory relying on phase transitions in chalcogenide glasses has been widely used for optical data storage, beyond that, weak optical nonlinearities have hindered the development of low‐power bistable devices. This work reports a new type of volatile optical bistability in a hybrid nano‐optomechanical device, comprising a pair of anchored nanowires decorated with plasmonic metamolecules. The nonlinearity and bistability reside in the mechanical properties of the acoustically driven nanowires and are transduced to the optical response by reconfiguring the plasmonic metamolecules. The device can be switched between bistable optical states with microwatts of optical power and its volatile memory can be erased by removing the acoustic signal. The demonstration of hybrid nano‐optomechanical bistability opens new opportunities to develop low‐power optical bistable devices.

## Graphical abstract

Optical memory is needed for all‐optical data storage and processing, but weak optical nonlinearities make low‐power optical memory states (bistability) difficult to obtain. This work shows how low‐power optical bistability can be derived from mechanical nonlinearity in a nano‐optomechanical structure consisting of mechanically nonlinear nanowires decorated with plasmonic metamolecules.

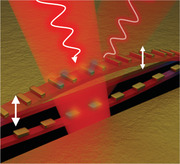

## 1 Search for Bistability in Photonics

A nonlinear system qualifies as bistable if it can exhibit two distinct output levels for the same level of input, depending on its history of excitation. While in electronics bistable devices such as flip‐flops are key components of circuits at all levels of integration, optical bistable devices have not yet reached the speed and energy consumption needed for wide application in signal processing and routing in all‐optical networks and photonic circuits.^[^ ^1^ ^]^

Although an optically driven anharmonic oscillator can exhibit a bistable response, in practice, the nonlinearity of optical electrons is not strong enough to reach bistability at light intensities below the optical breakdown of the medium. For this reason, most bistable systems in optics consist of a nonlinear or gain medium with an external^[^ ^2^ ^]^ or internal^[^ ^3^ ^]^ feedback mechanism. The earliest examples of volatile optical bistability include dispersive and absorptive transmission bistability in sodium vapor,^[^ ^2a^ ^]^ GaAs,^[^ ^2b^ ^]^ and other materials with Kerr nonlinearity in an optical cavity. Since then, optical bistability has been reported in photonic crystal microresonators,^[^ ^4^ ^]^ resonant nonlinear metamaterials,^[^ ^5^ ^]^ ring resonators,^[^ ^6^ ^]^ partly freestanding waveguide resonators,^[^ ^7^ ^]^ and lasers,^[^ ^8^ ^]^ where higher‐Q resonators enable lower‐power bistability at the cost of reducing optical bandwidth. An alternative approach to nonvolatile optical bistability involves materials undergoing light induced structural phase transitions, for instance chalcogenide glasses,^[^ ^9^ ^]^ vanadium oxides,^[^ ^10^ ^]^ or polymorphic gallium.^[^ ^11^ ^]^ Here, structural phase changes induced by heat of absorbed light modify the optical properties and provide the necessary conditions for optical bistability. Rewritable data storage optical disks with a silver‐indium‐antimony‐tellurium alloy active layer exploit this form of bistability.

**Figure 1:**
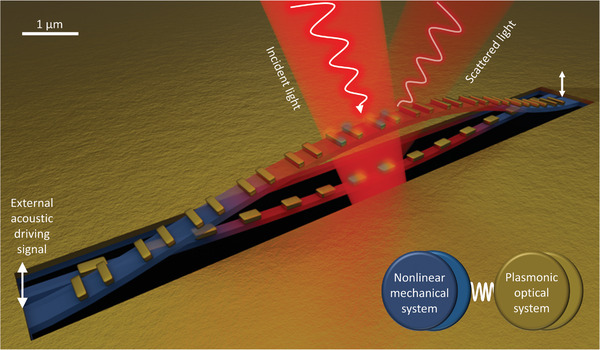
Optomechanical bistable system. Nanowires with anchored ends are nonlinear mechanical oscillators that can be driven into the bistable regime. Plasmonic metamolecules decorating a nanowire pair transduce the bistable oscillation into bistable optical properties and facilitate optical control of the bistability through light absorption (heat and thermal expansion). The bistability of the coupled system occurs at mechanical resonance frequencies and may be controlled either by the external acoustic driving signal (mechanical vibrations of the device) or by the power of incident light that tunes the temperature‐dependent resonance frequencies of the nanowires through optical heating.

In this paper we report an artificial nanostructure, a fundamental building block of photonic metamaterial,^[^ ^12^ ^]^ that exploits coupling between optical and mechanical subsystems to achieve optical bistability. This nanostructure is an array of plasmonic resonators with coupled parts of the resonators held on parallel nanowires cut from a flexible dielectric nanomembrane (**Figure** **1**). The nanowire mechanical subsystem of this device is highly nonlinear and can easily be driven to a bistable response. This bistable response can be controlled optically, by the heat dissipated from light absorbed by the plasmonic resonator optical subsystem. A light‐induced temperature increase expands the nanowires, tuning their resonant frequencies of oscillation, which drives considerable changes in the optical properties of the plasmonic resonators (metamolecules) split between the nanowires, providing conditions for optical bistability. We show that such a hybrid nano‐optomechanical system can be driven to a bistable response by picowatt acoustic signals and switched between bistable optical states with microwatt optical signals and ≈20 nJ of optical energy.

## 2 Observation of Bistability in a Nano‐Optomechanical System

The optically bistable nanostructure reported in this work consists of two coupled subsystems, a plasmonic subsystem that forms the optical response and a nanomechanical subsystem, the main source of nonlinearity. The nanomechanical subsystem is a pair of 50 nm thick and 16 µm long silicon nitride nanowires anchored to a silicon frame. The nanowires support an array of fourteen Π‐shaped gold nanorod plasmonic metamolecules 800 × 800 nm in size, parts of which are located on different nanowires (Figure S1, Supporting Information). The structure was fabricated by focused ion beam milling (FEI Helios 600 NanoLab) of a 50 nm thick, 16 µm × 400 µm rectangular, low stress (<250 MPa) silicon nitride membrane (Norcada Inc.) that was covered with a 50 nm gold film by thermal evaporation and supported by a 5 × 5 mm^2^ silicon frame of 200 µm thickness. The sample is mounted on a piezoelectric transducer with a central through‐hole (Thorlabs) acting on the silicon frame that supports the metamaterial nanowires (Figure S2, Supporting Information). The sample is actuated perpendicular to the plane of the structure by the force of inertia, when the transducer is driven by a voltage $U=\frac{\hat{U}}{2}\;\left(1+\sin\omega t\right)$, where $\hat{U}=0.6\;V_{p-p}$ unless specified otherwise. All experiments on optical scattering by the double nanowire structure were performed with normally incident radiation of a 1310 nm diode laser polarized parallel to the nanowires, with a full width at half maximum spot size of about 5 µm. Below, the optical power *P* incident on the nanowire pair is calculated assuming that the pair's total footprint is 16 µm × 800 nm. Back and forward scattered laser light in the direction normal to the sample plane were detected with photodetectors (New Focus Inc. 1811) and a lock‐in amplifier (Zurich Instruments UHFLI). Light modulation depth is given as peak‐to‐peak value relative to the average level. As is common for micro‐ and nanomechanical devices,^[^ ^13^ ^]^ the nanowire sample was placed in a vacuum chamber at 4 × 10^−3^ mbar pressure to reduce air‐damping of mechanical oscillations.

The plasmonic subsystem (i.e. the array of metamolecules) is resonant at the laser wavelength of 1310 nm. The resonance arises from coupling of a “bright” dipole mode in the larger nanorod and an anti‐symmetric “dark” mode in the pair of smaller nanorods and it enhances optical absorption and dispersive optical properties of the plasmonic subsystem (Figure S3, Supporting Information). The main out‐of‐plane flexural mechanical resonances of the wider and narrower nanowires occur at $\omega_{0}^{\prime }$ = 2*π* × 1.98 MHz and $\omega_{0}^{\prime \prime }$ = 2*π* × 1.53 MHz, respectively. They were identified as peaks in the power spectral density of light backscattered from the nanostructure due to thermal (Brownian) oscillations.^[^ ^14^ ^]^

**Figure 2:**
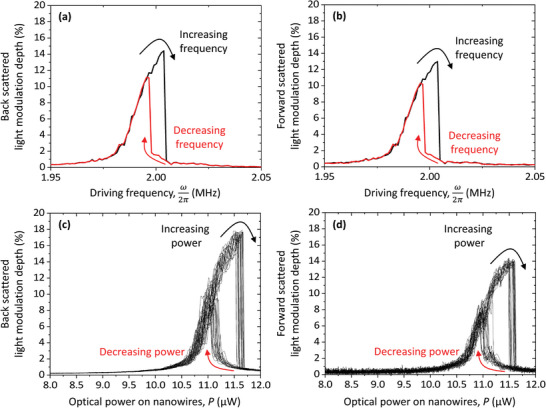
Acoustical and optical control of bistability. a,b) Modulation depth of a) back and b) forward scattered light for increasing (black) and decreasing (red) acoustic excitation frequency $\frac{\omega }{2\pi }$ at *P* = 11.3 µW optical power incident on the nanowire pair. c,d) Modulation depth of c) back and d) forward scattered light for fixed acoustic frequency $\frac{\omega }{2\pi }$ = 2.00 MHz and cycled optical power *P* (19 cycles).

The oscillations of the double‐clamped nanowires become highly nonlinear and enter the bistable regime when the span of flexural oscillations increases. This was observed by monitoring the amplitudes of modulation of light scattered on the sample in the backward and forward directions when increasing the sinusoidal AC voltage that drives the piezo actuator, Figure S4 (Supporting Information). Characteristically, the Lorentzian bell‐shaped resonance observed at low driving voltages (Figure S4a, Supporting Information) evolves with increasing driving amplitude into an asymmetric resonance with a sharp high frequency wing (Figure S4e, Supporting Information). The resonance shows hysteresis: the shape of the resonance curve depends on the scanning direction of the actuator driving frequency, Figure S4 (Supporting Information) and **Figure** **2**a,b. This hysteresis is the main feature of optical bistability: depending on the history of excitation, for the same input, the nanostructure exhibits two distinctly different optical output levels, e.g. at the driving frequency of 2.00 MHz the observed optical modulation depth can be either 14% or 1.0%.

To demonstrate optical control of the hysteresis cycle we keep the driving frequency fixed at 2.00 MHz while the incident laser power is linearly ramped up and down between 0 and 12.2 μW with a period of *T* = 1 s, Figure 2c,d. A hysteresis loop forms in between 10.9 and 11.7 µW of optical power incident on the nanowire pair. During cycling of the optical power, the bistable states remain robust across an optical power range of 0.3 µW, probing of which takes 12 ms (corresponding to 24 000 nanowire oscillations).

**Figure 3:**
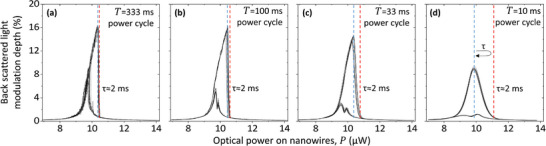
Optical bistability dynamics. Modulation depth of light back scattered on the metamaterial nanowire pair when the incident optical power is ramped up and down linearly between 6.8 and 14.2 µW with periods *T* from a) 333 ms to d) 10 ms (12 cycles each). Dashed lines indicate the maximum modulation (blue) and a 95% drop (red), and the acoustic excitation frequency is fixed at $\frac{\omega }{2\pi }$ = 2.00 MHz.

To study the transient dynamics of switching between the two metastable states we linearly ramped the laser power incident on the nanowires up and down between 6.8 and 14.2 µW with different periods *T* from 333 to 10 ms, **Figure** **3**. Here, the switching time *τ* can be evaluated as the time needed to change the laser power from the value corresponding to the peak of the higher branch of the hysteresis loop (blue dashed line) to the power level corresponding to the landing point on the lower branch (red), and back to the initial laser power on the lower branch (blue). For all scanning periods the switching time is approximately 2 ms.

Regarding endurance, we did not observe any noticeable degradation of the nanowire device during many hours of acoustic driving and optical illumination. The repeatability of switching between the bistable states is illustrated by many switching cycles shown in Figures 2 and 3.

## 3 Underlying Mechanisms of Bistability

The strong influence of the acoustic response of the nanowires on their optical scattering is the most important feature of the bi‐nanowire design which allows the translation of mechanical bistability of the nanowires into the hysteresis of their collective optical response. The resonant optical properties of the array of Π‐shaped metamolecules depend strongly on the mutual positions of the nanowires as one nanorod is located on the narrower nanowire and the other two are located on the wider nanowire.^[^ ^15^ ^]^

When the nanowires oscillate, driven by the piezo actuator, the mutual shift between the nanowires in the out‐of‐plane direction, and thus between the nanorods of the metamolecules, oscillates at the driving frequency, causing the modulation of light scattered on the nanostructure. The fundamental frequencies of the wider and narrower nanowires are different  ($\omega_{0}^{\prime }=2\pi \times 1.98\;\mathrm{MHz}$ and $\omega_{0}^{\prime \prime }=2\pi \times 1.53\;\mathrm{MHz}$), and are separated by more than the combined width of the resonances: $\omega_{0}^{\prime }-\omega_{0}^{\prime \prime }\gg \Gamma^{\prime }+\Gamma^{\prime \prime }$. Here,  Γ′ = 2*π* × 3.18 kHz and  Γ′′ = 2*π* × 3.21 kHz are the widths of the resonances at low amplitude of actuation (Figure S4a, Supporting Information). Although the external actuation drives the nanowires coherently, at the same frequency, the phase delay between their displacements depends on the detuning of the frequency of the driving force from the resonances, and, in the nonlinear regime, on its amplitude. For example, if the driving frequency is resonant with one of the nanowires, and off‐resonance with the other, the phase difference between their oscillations is ≈$\frac{\pi }{2}$.

We argue, that the bistability observed in our experiments is caused by nonlinearities of the nanomechanical subsystem of the nanostructure. Here, dynamics of the nanowire out‐of‐plane displacement *x* can be described by the damped nonlinear Duffing equation $\ddot{x}+\Gamma \dot{x}+\omega_{0}^{2}x+a\;\omega_{0}^{2}\;\frac{x^{3}}{h^{2}}=\frac{G}{m}\;\cos\left(\omega t\right)$,  where *ω*_0_ and Γ are the frequency and damping parameter of the main out‐of‐plane mode of the oscillation and *G* is the amplitude of the force acting on the nanowire with mass *m* when its supporting frame is shaken by the piezo actuator at frequency *ω*. The nonlinear term proprotional to *x* ^3^ accounts for stretching of the nanowire clamped at both ends during the oscillations. Such nonlinearity is known as geometric nonlinearity. It becomes significant when the amplitude of the oscillation approaches the thickness *h* of the nanowire. According to the Euler–Bernoulli theory of motion of a thin beam, the coefficient *a* of the nonlinear term depends on the vibrational mode and shape of the nanowire cross‐section and for the fundamental out‐of‐plane oscillation mode and a rectangular profile *a*  = 0.72.^[^ ^16^ ^]^ The amplitude of the driven Duffing oscillator increases with driving force, until it reaches the regime of bistability at the critical amplitude $x_{c}\approx 1.46\;\frac{h}{\sqrt{Q}}$.^[^ ^17^ ^]^ Here $Q=\frac{\omega_{0}}{\Gamma }$ is the quality factor of the nanowire oscillator. For the wider nanowire with Q = 622 and effective thickness *h* = 74 nm, the critical amplitude for the onset of bistable behavior can be estimated as *x_c_* ≈ 4.3 nm.

The magnitude of mechanical oscillations of the nanowire at the onset of bistable behavior observed in the experiment can be evaluated by comparing amplitudes of modulation of the scattered light intensity caused by the oscillations induced by the piezo actuator and by thermal movement of the nanowires at temperature *T*. The root mean square amplitude of thermal nanowire movements at the resonance $\left\langle x_{T}\right\rangle =\frac{1}{\omega_{0}}\;\sqrt{\frac{k_{B}T}{m\;}}$ is derived from the Langevin theory,^[^ ^18^ ^]^ that uses the same damped oscillator model, but with the nonlinear term neglected and the driving force of amplitude *G* replaced by the time‐dependent thermal force $\sqrt{4\pi k_{B}\;T\Gamma \;m}$ · *N*(*t*) related to the damping parameter Γ through the fluctuation‐dissipation theorem. Here, *N*(*t*) is normalized white noise.

The onset of bistability (observed at the piezo actuator driving voltage of 0.2 V, Figure S4b, Supporting Information) corresponds to 4% peak‐to‐peak modulation depth for light backscattered by the externally driven oscillator, while 0.05% root mean square modulation is induced by thermal movement of the nanowire. At *T* = 300 K the root mean square amplitude of thermal movement of the wide nanowire can be evaluated as 〈*x_T_* 〉 = 198 pm (effective mass *m* = 0.65 pg). Correspondingly, the 4% modulation observed at the bistable regime corresponds to a 15.8 nm peak‐to‐peak of nanowire oscillatory movement. This is within a factor of two of 2*x_c_* ≈ 8.6 nm peak to peak oscillatory movement anticipated from the Duffing model. The simplifying assumption of a rectangular nanowire shape, some uncertainty regarding the exact onset of bistability and the neglection of the nonresonant nanowire's oscillation may contribute to the imperfect match.

The restoring force acting on the nanowire is nonlinear *F* = *kx* + *µ* *x* ^3^ , where the spring constant of the wide oscillator $k=m\omega_{0}^{2}=0.10\;N\;m^{-1}$ and $\mu =\frac{am\omega_{0}^{2}}{h^{2}}$ = 1.3 × 10^13^ N m^−3^. When a fully opened hysteresis loop is observed, the amplitude of oscillation is at maximum *x*_max_ ≈ 28 nm. At *x*_max_ the linear part of the nanowire's restoring force *kx* ≈ 2.8 nN, while the nonlinear restoring force *µ* *x* ^3^ ≈ 0.29 nN. Nevertheless, at the high *Q* resonance the small nonlinear component of the restoring force is sufficient to provoke bistability. The energy accumulated in the nanowire motion can be evaluated as $E=\frac{k\;x_{\max}^{2}}{2}+\frac{\mu \;x_{\max}^{4}}{4}\approx 41\;\mathrm{aJ}$, where the linear contribution is dominant. Therefore, the mechanical energy dissipation rate $P_{m}\approx \frac{\omega_{0}E}{Q}=0.82\;\mathrm{pW}$. This power must be supplied continuously by the piezo actuator to the nanowire to operate the bistable device. The optical bistability/memory function will be erased by switching off the acoustic actuation, making this an example of erasable volatile memory.

Assuming a dominant linear contribution to the oscillator energy, and that the amplitude of oscillations at the bistable regime *x*_max_ ≅2*x*_c_, the acoustic power needed to operate in the bistable regime $P_{m}\approx \;\frac{\omega_{0}E}{Q}\propto \;\frac{m\;\omega_{0}^{3}h^{2}}{Q^{2}}$ increases with mass *m*, thickness *h*, and frequency of oscillations *ω*_0_ of the nanowire and decreases with its quality factor *Q*.

The ability to control the hysteresis loop with external optical stimulation could rely on two different mechanisms. The first one is the emergence of an optical force between the light induced oscillating dipoles of the Π‐shaped metamolecules. In terms of the Duffing model, it will be a time‐dependent driving force on the right side of the equation of motion of the nanowire. At light intensities used in the experiment the light‐induced dipole forces are weak, reaching only sub‐pN levels. Indeed, when the nanowire is illuminated with the light intensity needed to achieve hysteresis control *I* = 3 × 10^6^ W m^−2^, such force $F\;=\frac{\delta E}{\;x_{c}}$, per Π‐shaped metamolecule split between two nanowires, can be evaluated from the difference of electromagnetic energies of the metamolecule *δE* ≈ 6.5 × 10^−23^ J at two mutual positions different by *x*_c_ ≈ 4 nm, calculated numerically from the field distributions at the metamolecule, giving *F* ≈ 16 fN per metamolecule and 0.22 pN for the nanowire supporting 14 metamolecules, which is three orders of magnitude weaker than the restoring force of the nanowire at the same displacement.

**Figure 4:**
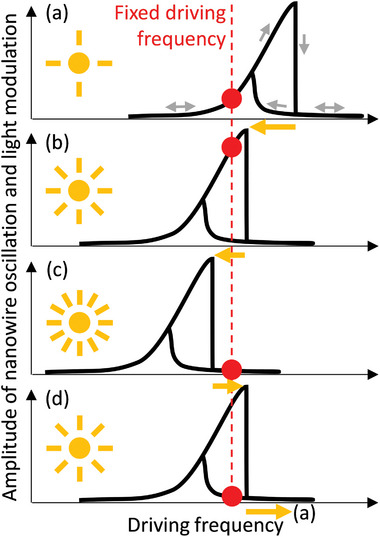
Mechanism of optical control of optomechanical bistability. The nanowire device is driven acoustically to a highly nonlinear regime in which hysteresis of the nanomechanical response can be observed. The amplitude of modulation of light scattered on the device in backward and forward directions follows the amplitude of nanowire oscillation. a) The acoustic driving frequency is set (red) below the mechanical resonance while optical excitation (yellow sun) is at a low level. b) With increasing optical power thermal expansion reduces the stress in the nanowire and the hysteresis shifts (yellow arrow) towards lower acoustic frequencies. The upper branch of the hysteresis loop is followed. A high amplitude of optical modulation is achieved. c) With further increase of optical power, the hysteresis cliff shifts beyond the fixed acoustic driving frequency: the nanowire switches to low amplitude oscillation. d) Reducing optical power allows the nanowire to cool and the acoustic hysteresis shifts towards higher frequencies. The nanowire oscillation amplitude follows the lower branch of the hysteresis loop. A low amplitude of optical modulation is observed. Returning to the low optical power restores situation (a).

The second mechanism of optical control of the oscillating nanowires is parametric. It results from the change of a nanowire's natural frequency of oscillation caused by its thermal expansion due to a light‐induced temperature increase, as illustrated by **Figure** **4**. For central illumination, the average temperature increase Δ*T* of a nanowire above the ambient temperature *T*_0_ can be evaluated as $\Delta T\;=\frac{T_{\max}-T_{0}}{2}\approx \;\frac{\xi P^{\prime }L}{8\kappa \mathrm{wh}}$,^[^ ^17^ ^]^ where *T*_max_ is the central temperature, *L*, *w*, *h*  are length, width, and thickness of the nanowire,  *P*′ = 0.53 *P* is the laser power falling on it, *ξ* is the absorbed fraction of incident power (which is controlled by the metamolecule design), and *κ* is the nanowire's thermal conductivity. For *P*′ = 5 µW, *ξ* = 0.5, *L* = 16 µm, *w* = 280 nm, *h* = 74 nm, and *κ* = 31 Wm^−1^K^−1^, the temperature increase is Δ*T* ≈ 8 K. Here, the nanowire cooling time can be evaluated as $\Theta \;=\frac{L^{2}C}{8\kappa }\approx \;2.2$µs, where *C* = 2.2 J cm^−3^K^−1^ is the volume heat capacity.

To verify that the change of the natural frequency of the nanowire oscillator is caused by thermal expansion, we compare the experimentally observed laser‐induced rate of frequency shift *χ*_exp_ = $\frac{\Delta \omega_{0}^{\prime }}{2\pi \;\Delta P^{\prime }}=\;-\frac{60\;\mathrm{kHz}}{\mu W}$ with the shift calculated from the thermal expansion model. The mechanical resonace frequency of a doubly clamped nanowire *ω*_0_ depends on its stress *σ*  as follows: $\omega_{0}\;\left(\sigma \right)=\;2.06\;\;\frac{\pi \;h}{L^{2}}\sqrt{\frac{\eta }{\rho }\times \left(1+\frac{\sigma L^{2}}{3.4\eta h^{2}}\right)}$, where *η* is the Young's modulus and *ρ* is the nanowire density. Solving this equation with *ω*_0_ = 2*π* × 2.35 MHz returns the value of stress in the absence of illumination, *σ*_0_ = 21 MPa. The laser heating reduces the tensile stress of the nanowire proportionally to the temperature increase *σ* = *σ*_0_ − *αη* × Δ*T*, where  *α* = 4 × 10^−6^ K^−1^ is the thermal expansion coefficient of the nanowire.^[^ ^15^ ^]^ As a result, the nanowire's natural frequency $\omega_{0}^{\prime }$ reduces with increase of the laser power  $\Delta \omega_{0}^{\prime }\approx \;\;\frac{\partial \omega_{0}^{\prime }}{\partial T}\;\Delta T\;=\;-\omega_{0}^{\prime }\;\times \frac{\alpha }{6.8{\left(\frac{h}{L}\right)}^{2}+\frac{2\sigma_{0}}{\eta }}\;\times \;\frac{\xi P^{\prime }L}{8\kappa \mathrm{wh}}$. The frequency shift $\chi_{\mathrm{thermal}}=\;-\frac{41\;\mathrm{kHz}}{\mu W}$ calculated from this formula is comparable to the experimentally observed laser‐induced rate of frequency shift, thus confirming that the thermal mechanism of the light‐induced frequency shift dominates. For the above estimates we derived the effective thermal conductivity *κ* = 31 W m^−1^K^−1^ of the silicon nitride nanowire decorated with gold nanorods from a thermal circuit model and used volume weighted average values for the Young's modulus, density, and thickness of the nanowire (*η* = 202 GPa, *ρ* = 8209 kg m^−3^, *h* = 74 nm), see Table S1 (Supporting Information).

Finally, the switching dynamics observed with shortening cycles of optical power modulation (Figure 3) show that a switching operation can be achieved in *τ* ≈ 2 ms which is much longer than the nanowire cooling time $\Theta \;=\frac{L^{2}C}{8\kappa }\approx 2.2$ µs and shall be compared with the nanowire's linear oscillator relaxation time $\delta \approx \frac{2}{\Gamma }=\;0.1\;\mathrm{ms}$, i.e., the time it takes for the oscillation amplitude to fall to 1/e. Considering that a 95% drop (as in Figure 3) will take 3*δ* and the return to the initial power, such a switching operation may be expected to take 6*δ* ≈ 0.6 ms. This linear estimate is smaller than the experimentally observed switching time *τ*, as it neglects that in the highly nonlinear regime of bistability the initial relaxation rate decreases as a function of the excitation energy for the oscillator coupled to a dissipative bath.^[^ ^19^ ^]^ The optical energy required to switch between the bistable states of a nanowire pair in our experiments can be evaluated from the optical power *P* = 11 µW incident on the nanowire pair and switching time *τ* ≈ 2 ms as ≈ 22 nJ.

Optical control of states of the bistable mechanical oscillator can be achieved when $|\Delta \omega_{0}^{\prime }|\approx \;\Gamma^{\prime }$, i.e., $\Delta P^{\prime }\approx \;\left(6.8{\left(\frac{h}{L}\right)}^{2}+\frac{2\sigma_{0}}{\eta }\right)\times \frac{8\kappa \mathrm{wh}}{\alpha \xi \mathrm{LQ}}=\;80\;\mathrm{nW}$, which corresponds to a change of the optical power incident on the nanowire pair of Δ*P* ≈ 150 nW. This matches the range of optical powers at which the abrupt transition is observed (Figure 2c,d) and suggests that, in principle, a switching operation is conceivable with as little as Δ*P* *τ* ≈ 300 pJ of optical energy. In a stressed membrane, typically $6.8{\left(\frac{h}{L}\right)}^{2}\ll \;\frac{2\sigma_{0}}{\eta },$ and then $\Delta P^{\prime }\approx \frac{16\kappa \mathrm{wh}\sigma_{0}}{\alpha \;\xi \;L\;Q\;\eta }$. Either way, the optical power (or power change) required for switching between the bistable states increases with increasing thermal conductivity *κ*, width *w*, thickness *h*, and stress *σ*_0_ of the mechanically bistable nanowire and decreases with increasing thermal expansion coefficient *α*, absorption coefficient *ξ*, length *L*, Young's modulus *η* of the nanowire, and quality factor *Q* of the mechanical mode.

We shall consider how the bistability may be optimized. As the switching time $\tau \propto \;\frac{Q}{\omega_{0}}$ is controlled by the mechanical response time of the nanowires, faster switching may be achieved by reducing the *Q*‐factor of the mechanical resonance (e.g., increased air damping) or, assuming the same *Q*, by using (e.g., shorter) nanowires with higher resonance frequency *ω*_0_. However, the reduced time *τ* comes at the cost of increased optical power Δ*P* required for switching. The switching energy Δ*P* *τ* will not be affected by damping as the switching time and power have inverse dependencies on *Q*. In the limit of nanowires with low stress, $\omega_{0}\propto L^{-2}$, $\Delta P\propto \kappa Q^{-1}L^{-3}$, $\Delta P\tau \propto \kappa L^{-1}$ and for high stress $\omega_{0}\propto L^{-1}$, $\Delta P\propto \kappa Q^{-1}L^{-1}$, ΔPτ∝κ. Thus, shorter nanowires with low stress require more energy to switch. The optical power and energy requirements for switching can be decreased without affecting *τ* by reducing the thermal conductivity *κ* of the nanowires (e.g., by removing the gold at their ends), provided that the cooling timescale Θ remains short compared to the mechanical relaxation time *δ*.

Even without such optimization, the bistability was observed at optical power *P* = 11 µW incident on a nanowire pair and should in principle require as little as Δ*P* ≈ 150 nW, making it substantially lower power than integrated bistable systems based on thermal nonlinear processes of silicon^[^ ^6b^ ^]^ and carrier generation (≈mW),^[^ ^20^ ^]^ also with air gaps providing thermal insulation of a free‐standing silicon ring (≈0.1 mW),^[^ ^6a^ ^]^ as well as optomechanical ring structures (>0.1 mW)^[^ ^6^, ^21^ ^]^ and freestanding waveguides (≈1 mW)^[^ ^7^ ^]^ driven by optical forces, and even low volume photonic crystal nanocavities (≈µW).^[^ ^4b^ ^]^ Such systems generally rely on high‐*Q* optical resonances to minimize their power requirements or for optomechanical transduction, which limits their operation to a narrow wavelength range (<1 nm). We note that much lower power optical bistability can be achieved under extreme conditions, e.g. optical bistability at 20 pW has been reported in ultracold gases (≈20 µΚ)^[^ ^22^ ^]^ and with extremely small optical bandwidth (<1 fm). In contrast, the room temperature optical bistability reported here can be expected to offer large optical bandwidth (hundreds of nanometers), which may be estimated from the width of the structure's low‐*Q* absorption resonance (Figure S3, Supporting Information).

The role of the plasmonic structure's resonance is twofold. Firstly, the optical power required for switching is inversely proportional to the fraction of light energy absorbed by the nanostructure. As illustrated by the resonant absorption spectrum of an array of the metamaterial nanowires (Figure S3, Supporting Information), the resonance makes optical heating of the nanowires more efficient by increasing absorption, allowing the bistability to be controlled at lower optical power levels. Secondly, the resonance makes the optical properties of the plasmonic structure highly sensitive to relative displacement of the plasmonic nanorods.^[^ ^15^ ^]^ It is expected that larger light modulation can be achieved through optimization of the metamolecule sensitivity to mutual displacement of its parts.

As the observed bistable states are characterized by different amplitudes of optical signal modulation at the frequency of nanowire oscillation, they can be used to represent information. Indeed, the two bistable states in Figure 2c correspond to (1.25 ± 0.21) % and (15.1 ± 1.0) % of modulation (in a 0.1 µW optical power range centered at 11.4 µW). As the states are separated by many standard deviations, they are highly distinguishable, implying vanishingly small bit error rates in digital applications.

## 4 Conclusions

In conclusion, we have demonstrated that a hybrid nano‐optomechanical system can exhibit a low power bistable optical response. We have shown that bistable states arising from the mechanical nonlinearity of nanowires give rise to bistability of optical properties, and that the incident light can control switching between these states.

## Conflict of Interest

The authors declare no conflict of interest.

## Supporting information

Supporting Information

## Data Availability

The data that support the findings of this study are openly available in the University of Southampton ePrints research repository at https://doi.org/10.5258/SOTON/D2077.
