# Supplementary material for: Microwatt Volatile Optical Bistability via Nanomechanical Nonlinearity
Source: Adv Sci (Weinh). 2023 Apr 26;10(18):2300042. doi: 10.1002/advs.202300042 (PMC10288264; doi:10.1002/advs.202300042)
Supplement: Supplementary file 1 — Supporting Information [file ADVS-10-2300042-s001.pdf]

ADVANCED SCIENCE
Open Access

Supporting Information

for *Adv. Sci.*, DOI 10.1002/advs.202300042

Microwatt Volatile Optical Bistability via Nanomechanical Nonlinearity

Dimitrios Papas, Jun-Yu Ou, Eric Plum and Nikolay I. Zheludev*

Supporting Information

Microwatt volatile optical bistability via nanomechanical nonlinearity

Dimitrios Papas, Jun-Yu Ou, Eric Plum, Nikolay I. Zheludev*

Table S1. Thermal and mechanical properties of silicon nitride and gold

	Silicon nitride (SiN)	Gold (Au)
Young's modulus, η (GPa)	260	77
Density, ρ (kg m^{-3})	3100	19300
Thermal expansion coeff. α (K^{-1})	2.8 x 10^{-6}	14.2 x 10^{-6}
Thermal conductivity, κ ($Wm^{-1}K^{-1}$)	318	26
Volumetric heat capacity, C (J $cm^{-3}K^{-1}$)	2.1	2.5

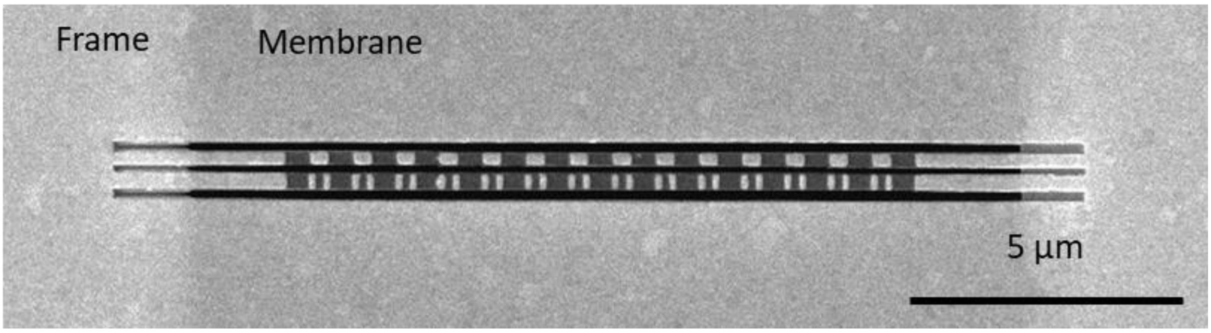

Figure S1. Bistable metamaterial nanowires. SEM image of a pair of metamaterial nanowires consisting of silicon nitride beams decorated with gold plasmonic nanorods.

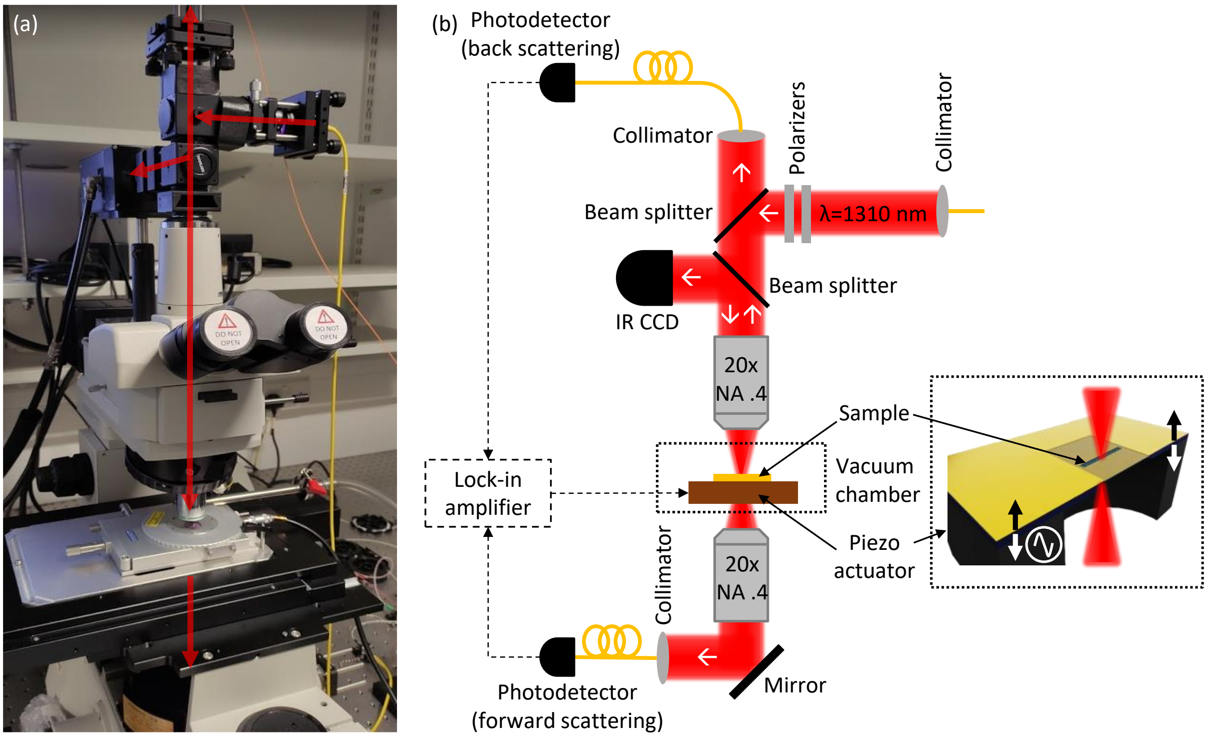

Figure S2. Experimental setup used for measurements of optical bistability via nanomechanical nonlinearity. (a) Photo and (b) schematic of the setup.

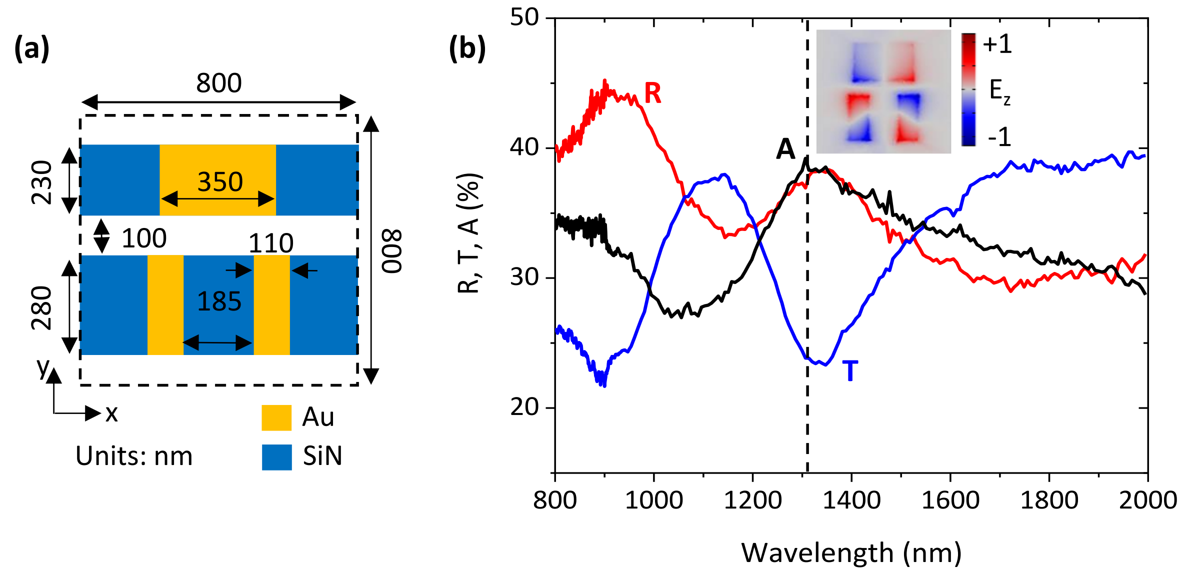

Figure S3. Metamaterial geometry and optical properties. (a) Unit cell dimensions. (b) Measured reflection (red), transmission (blue) and absorption (black) spectra of a nanomechanical metamaterial array consisting of many of the metamaterial nanowire pairs shown in Figure S1. The inset shows the simulated E_z field distribution 10 nm above the plasmonic structure for incident radiation of 1310 nm wavelength. The electromagnetic simulation of the resonant optical mode was performed by finite element method modelling (COMSOL Multiphysics 5.3a) of a single unit cell with periodic boundary conditions. Light is normally incident and polarized parallel to the silicon nitride nanowires.

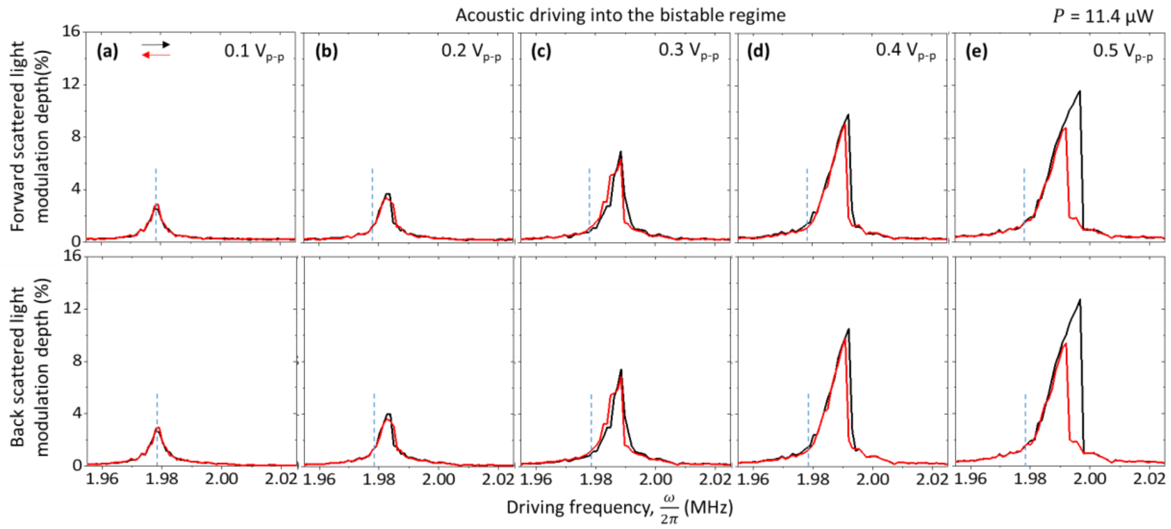

Figure S4. Onset of optomechanical bistability. (a-e) Modulation depth of light forward (top) and back (bottom) scattered on metamaterial nanowires driven to oscillate at increasing (black) and decreasing (red) mechanical frequencies with different driving amplitudes.
